# Cross-species anxiety tests in psychiatry: pitfalls and promises

**DOI:** 10.1038/s41380-021-01299-4

**Published:** 2021-09-24

**Authors:** Dominik R. Bach

**Affiliations:** Wellcome Centre for Human Neuroimaging and Max Planck UCL Centre for Computational Psychiatry and Ageing Research, University College London, London, UK

**Keywords:** Depression, Neuroscience

## Abstract

Behavioural anxiety tests in non-human animals are used for anxiolytic drug discovery, and to investigate the neurobiology of threat avoidance. Over the past decade, several of them were translated to humans with three clinically relevant goals: to assess potential efficacy of candidate treatments in healthy humans; to develop diagnostic tests or biomarkers; and to elucidate the pathophysiology of anxiety disorders. In this review, we scrutinise these promises and compare seven anxiety tests that are validated across species: five approach-avoidance conflict tests, unpredictable shock anticipation, and the social intrusion test in children. Regarding the first goal, three tests appear suitable for anxiolytic drug screening in humans. However, they have not become part of the drug development pipeline and achieving this may require independent confirmation of predictive validity and cost-effectiveness. Secondly, two tests have shown potential to measure clinically relevant individual differences, but their psychometric properties, predictive value, and clinical applicability need to be clarified. Finally, cross-species research has not yet revealed new evidence that the physiology of healthy human behaviour in anxiety tests relates to the physiology of anxiety symptoms in patients. To summarise, cross-species anxiety tests could be rendered useful for drug screening and for development of diagnostic instruments. Using these tests for aetiology research in healthy humans or animals needs to be queried and may turn out to be unrealistic.

## Introduction

Anxiety disorders constitute a major part of the disease burden in mental health [1]. Current guidelines recommend treatment with psychotherapy, selective serotonin reuptake inhibitors (SSRI) or GABAergic medication [1]. Effectiveness of these interventions leaves room for improvement [1], motivating pre-clinical research in non-human animals. A popular approach relies on behavioural anxiety tests: simple and brief setups that elicit some form of adaptive avoidance behaviour, which is modified by anxiolytic drugs. Yet, a steady flow of anxiolytic drug discoveries in these tests [2] has not resulted in any clinical innovation for decades [1, 2]. A number of potential reasons for this stagnation have been prominently discussed. The first is the narrow scope of anxiety tests in relation to biological scenarios [3, 4], and their conceptual inadequacy in relation to clinical conditions [5–7]. However, even if the tests were conceptually adequate, behavioural control might still differ substantially between species as disparate as rodents and humans, which diverge in many aspects of their neurobiology, from receptor distribution to macroscopic structure of the neocortex [8], and ensuing human-specific cognition [9, 10]. To disentangle these potential shortcomings, several behavioural anxiety tests have been translated across species and to humans. Investigation across species may clarify whether or not humans and non-humans solve the same task differently (species differences), and whether or not behavioural control in the human version of the task resembles features of anxiety disorder (adequacy of the test). Three clinically relevant goals have been proposed in relation to this cross-species translation. The first goal is screening of potential anxiety treatments in pre-clinical human studies [10, 11]. A drug that reduces avoidance behaviour in animals could be ineffective in humans due to slight neurobiological differences between species. Such trivial translational failures could potentially be detected in a pre-clinical human test, before initiation of clinical trials. Secondly, if anxiety tests are sensitive to induced anxiety symptoms, then they could be developed into diagnostic tests or biomarkers based on observable behaviour [12]. The third goal is to find at least coarse evidence that healthy humans’ behaviour in the anxiety test is controlled by similar neural mechanisms as symptoms of anxiety disorder. This would validate the test, and support extrapolating a wealth of neurobiological findings in animal and human anxiety tests to symptoms of anxiety disorder [9, 13].

Indeed, cross-species anxiety tests have led to remarkable insights into the basic neurobiology and cognitive mechanisms of threat avoidance behaviour in healthy individuals. Expanding on the classic septo-hippocampal model established in the 1980s [14, 15], more recent research has highlighted the contribution of various additional brain regions to avoidance behaviour, such as amygdala [16–18], nucleus accumbens [19, 20], area 25 [21], as well as striatum and anterior cingulate cortex (ACC) [22–27]; how they communicate by neural oscillations [28–34]; the more fine-grained role of hippocampal subfields [35–39]; and the computational mechanisms that underly behaviour under threat [40–43] as well as their neural implementation [44]. Despite these fascinating insights, however, we argue that the field is yet to deliver on the three clinical promises. We suggest a focused research agenda to identify unrealistic goals, and to overcome obstacles to achieving more realistic ones.

## Status of anxiety tests

We consider three common categories of behavioural tests that induce an immediate and short-lasting phenotype of anxiety-like behaviour, and have been translated across species: approach-avoidance conflict (AAC) tests, unpredictable shock anticipation and social intrusion. We do not cover models that primarily focus on longer-lasting phenotypes; these have a different conceptual basis, as will become apparent below. Thus, we do not discuss behavioural PTSD models based on experimental stress, trauma or aversive memory (see, e.g., [45, 46] for review); and pharmacological and genetic manipulations (most of which are impossible to use in humans; see, e.g., [2, 5] for review).

Across medical conditions, pre-clinical paradigms are often grouped into screening tests and disease models [47]. Screening tests are used in drug-discovery research to predict clinical potential of novel compounds [2, 47]. They can be narrowly focused on identifying drugs that act on a particular receptor; and in this case they need not have any conceptual relation to the disease that is ultimately being treated, as long as they can predict the treatment response well. Anxiety tests are an example of such screening tests as many of them predict the clinical action of GABAergic drugs [48]. However, screening tests may also be predictive across drug classes, and in reverse they may be sensitive to manipulations that induce or worsen disease symptoms [4, 47]. If a test is sensitive to induced disease symptoms, then its human translation may be sensitive to the disease itself. Consequently, human translation of screening tests has been suggested as a way of developing diagnostic instruments [12], which could fill a current lack of ‘standardised cognitive and psychological measures’ [49] of mental health function. Furthermore, disease symptoms in many medical conditions (e.g., hypertension or diabetes) vary along a continuum without pre-defined boundaries. If that is also the case in psychiatry, as suggested recently [50], then such tests may be able to differentiate among clinically healthy humans or wildtype animals. Such properties have for example been suggested for cross-species tests of impulsivity [51].

Disease models, in contrast, are manipulations that create disease processes or symptoms in the laboratory. Three broad criteria serve to evaluate such disease models [47]. The first is predictive validity: all treatments that alleviate or worsen symptoms in the disease should have the same effect in the model, and vice versa, across different drug mechanisms [47]. The second criterion is phenomenology or face validity: the symptom-eliciting procedure, the elicited symptoms, the treatment response (e.g., its dynamics) and the underlying physiology should all be similar to what is observed in the disease [47]. Finally, the theoretical basis of the model should match the disease aetiology [47]; this is almost impossible to assess in psychiatry, where disease aetiology is unknown for many conditions.

Despite their established predictive validity for at least some drug classes, behavioural anxiety tests lack face validity as disease models [5, 48]. The behaviour-eliciting procedures are mostly unrelated to factors that induce or facilitate anxiety disorders. The elicited behaviour is short-lasting and forms part of wildtype animals’ standard repertoire; it is not a disease symptom. Clearly, the defining feature of anxiety disorders is not the adaptive situational avoidance of an aversive situation as in an anxiety test, but the lasting maladaptive avoidance of objectively harmless situations. This lack of face validity is in contrast to genetic, pharmacological or behavioural anxiety models that create longer-lasting phenotypes, with behavioural dynamics more closely related to anxiety disorders.

Nevertheless, it is often hoped that understanding normal behaviour in anxiety tests can tell us about disease mechanisms [9, 13]. This is based on the premise that ‘anxiety disorders represent an exaggerated activation of the normal fear response’ [5]. For example, the test might be a model not of the disease itself but of disease symptoms: while circumstances and neural mechanisms that elicit avoidance behaviour in patients and in the test might be very different, the neural control of that behaviour, once engaged, could be similar [48]. In this case, the test might allow identification of intervention targets for symptom attenuation. However, the only non-human evidence to date in favour of this premise is the effectiveness of GABAergic, and (sometimes) serotonergic drugs, in reducing avoidance behaviour in these tests as well as symptoms in clinical conditions. Here we review further human evidence for the assumption that the physiology of avoidance behaviour in anxiety tests, and anxiety disorder symptoms, is shared.

## Principles of cross-species translation

Most behavioural anxiety tests rely on tangible threats to the animal. For example, rodents must reasonably expect predation in an open field test—as in their natural habitat. In other tests, they are treated with strong electric shocks without propositional knowledge on the cause of the inflicted pain. A crucial limitation of human translation is that for ethical reasons most actual threats must be removed or alleviated. A plethora of research suggests that the brain may contain multiple decision-making systems [52], and that the human mind or brain may solve abstract, deliberate decisions different from implicit choices [53], in particular under threat [54, 55]. To corroborate that comparable decision-making systems are invoked when a test is translated across species [56], we apply the same criteria that are used to validate disease models. One is predictive validity: the same treatments that reduce anxiety-like behaviour in animals should reduce anxiety-like behaviour in the human test. This encompasses, for example, the well-known effects of GABAergic anxiolytics, or the anxiolytic effects of (ventral) hippocampus lesions in AAC tests. Another criterion is face validity. First, regarding the behaviour-eliciting procedure, the human paradigm should be similar to the rodent paradigm, with the limitation that threats must usually be converted to mild primary reinforcers [57] or to simulated threats (e.g., in serious computer games [58]). Secondly, is human behaviour in the test similar to animal behaviour? An obvious limitation is that several human tests are conceived as third-person view computer games with keyboard responses, which puts strong constraints on possibly observable behaviours within the setup. Hence, only a few paradigms can be evaluated in this respect. A third aspect is the physiology, which partly encompasses predictive validity: are similar physiological mechanisms involved across species in controlling anxiety-like behaviour? This relates for example to the increase in hippocampal theta and gamma power, or cross-regional oscillatory coupling, in AAC tests. Regarding the fourth aspect, treatment dynamics, there is a dearth of empirical data on anxiety tests, and as such it will not be considered further.

## Cross-species anxiety tests

The first category of cross-species paradigms considered here, AAC tests, comes in many different flavours. Generally, animals are exposed to supposedly conflicting motivations to approach an object or location, and to avoid it at the same time [59]. These motivations can be innate or learned in the experiment, and there are multiple ways of quantifying the animal’s response [59]. Beyond precisely modelling a specific animal test in humans, there is a plethora of human tests that involve some form of response conflict but do not relate to any specific animal setup. Here, we restrict the discussion to those cross-species paradigms that either directly reflect an animal test without reduction to an abstract computer task, or for which cross-species validity has been established with anxiolytics, hippocampal lesions or by direct replication of neural phenomena across species. This excludes human conflict paradigms that do not model a specific animal anxiety test in the first place [42, 43, 60–62], or that have been investigated with fMRI only, but no fMRI (or other tissue oxygenation) data exist for the animal version [63, 64]. Similarly, although stress and anxiety are closely interlinked [65], we exclude the large range of human stress tests that do not reflect animal paradigms, for example those that employ negative social feedback as stressor, such as public speaking paradigms [66–68].

### Elevated plus maze

In the elevated plus maze (EPM) [69] and its variants [59], a rodent is placed in an apparatus with narrow running tracks that are elevated above ground, and partly open, partly closed. Rodents will generally avoid the open arms, presumably due to an innate tendency to avoid height and open space. Benzodiazepines and partial serotonin 1A receptor (5HT1A) agonists increase open arm entries, while this has not been demonstrated for SSRIs [2]. Ventral hippocampus lesions more often than not increase open arm entries [70–75].

**Fig. 1:**
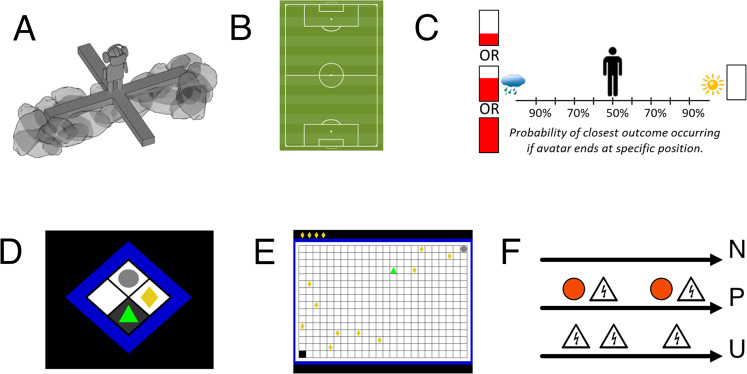
Human versions of the reviewed cross-species anxiety paradigms. **A** Elevated plus maze: mixed-reality setup in which human participants explore a plank over a deep drop and another one that rests on a rocky ground. **B** Open field test. Participants explore, in reality or virtual reality, a large space up to the size of a soccer pitch. **C** Approach-avoidance conflict decision test. In a lottery, participants can decide on their desired probability of a neutral outcome (depicted by sun) and of a reward (red bar) coupled with an aversive sensory experience (depicted by rain). **D** Approach-avoidance conflict ‘scoop & run’ test. Participants move an avatar (green triangle) outside a safe place and back to collect a financial reward token (yellow rhombus), under threat of being caught by a virtual predator (grey circle) and losing tokens. **E** Approach-avoidance conflict ‘stay & play’ test. Participant move an avatar (green triangle) on a 24 × 16 grid to collect multiple to financial reward tokens (yellow rhombi), under threat of being caught by a virtual predator (grey circle) and losing all tokens. **F** NPU test. In a predictable condition, aversive outcomes are always cued, whereas they appear at random in an unpredictable condition. In a neutral condition, no aversive outcomes occur. The social intrusion test (no illustration) quantifies children’s behavioural inhibition in social contexts. **A** is reproduced from ref. [76]. **B** was created by Hazaña17 (https://commons.wikimedia.org/w/index.php?curid=16311903) under CC BY-SA 3.0. **C** is provided by courtesy of Dr Robin Aupperle. **D**–**F** are the author’s own work.

Biedermann et al. [76] (Fig. 1A and Table 1) developed a human mixed-reality version of the EPM, in which a real plus maze provides haptic cues and is combined with virtual reality to generate visual experience. In this setup, participants preferentially sought out the closed arms. This tendency was reduced under the benzodiazepine lorazepam, and increased under the anxiogenic yohimbine [76]. In *N* = 100 participants, sex predicted anxiety-like behaviour [76]. In a related paradigm using virtual reality only, no data relevant for validation were reported [77]. The human EPM resembles features of virtual reality-based psychotherapy setups that are used to elicit height phobia symptoms in patients [78].

**Table 1:** Cross-species anxiety tests and their potential application.

Test (references: studies considered)	Cross-species translation		Translational application	
	Predictive validity	Face validity	Drug-screening test	Diagnostic test
Elevated plus maze (EPM) [76]	Lorazepam	Procedure: yes Behaviour: yes Physiology: N/A	GABAergic drugs, potentially anxiogenic drugs	Potentially (effects of anxiogenic substances, sex differences)
Open field test [81, 84, 86]	N/A	Procedure: yes Behaviour: yes Physiology: N/A	N/A	Potentially (differentiating clinical groups) No consistent relation to self-reported anxiety across samples
Operant conflict tests: AAC decision test [27, 57, 94, 95]	N/A	Procedure: partly Behaviour: partly Physiology: AAC fMRI signal in conflict > non-conflict trials (by comparison with a macaque version [27] that is itself validated with diazepam [24])	N/A	Potentially (sex differences)
Operant conflict test: AAC scoop & run test [18, 33, 39, 40]	HC lesions (by comparison with other approach-avoidance conflict tests)	Procedure: partly Behaviour: partly Physiology: HC gamma oscillations and theta HC-PFC synchronisation (by comparison with other approach-avoidance conflict tests)	N/A	Potentially
Mixed open field/operant conflict test: AAC stay & play test [11, 17, 58, 96]	GABAergic drugs (lorazepam, pregabalin, valproate), HC lesions (by comparison with other approach-avoidance conflict tests)	Procedure: partly Behaviour: partly Physiology: N/A	GABAergic drugs	Potentially (stable individual differences in behavioural disposition. No relation to self-reported anxiety in large sample
Unpredictable shock anticipation (NPU test) [107–111, 113–117]	Alprazolam	Procedure: yes Behaviour: yes Physiology: N/A	GABAergic drugs, SSRIs	Yes (distinguishes PTSD, SAD, SP, PD from HC, GAD, MDD, relates to symptom severity in trauma-exposed people)
Social intrusion test [118–126]	N/A	Procedure: yes Behaviour: yes Physiology: N/A	N/A	Yes (stable individual differences, heritability, risk factor for anxiety disorders)

Cross-species translation: predictive validity of anxiolytic procedures, and face validity of the test, with respect to the underlying animal model. Translation application: suitability as a drug-screening test, or as a diagnostic/psychometric test.

*N/A* no data available.

### Open field test

In the open field test [79], a rodent is placed into an arena of around 60 × 60 cm size [59] and free to explore it. Rodents typically avoid the centre of the arena (thigmotaxis), presumably due to an innate tendency to avoid predation associated with open spaces. Benzodiazepine and barbiturate receptor agonists more often than not reduce thigmotaxis [80]; this effect is increased by water or food deprivation and hence presumably due to an innate tendency to forage for food and water [80]. Conversely, benzodiazepine antagonists increase thigmotaxis [80]. However, some other clinically effective GABAergic anxiolytics such as triazolobenzodiazepines do not show consistent effects across studies [80]. Partial 5HT1A agonists such as the clinically effective buspirone show anxiolytic effects in the majority of studies [80], and so do SSRIs after repeated administration [2]. Ventral dentate gyrus is involved in controlling thigmotaxis [37] and lesioning it reduced thigmotaxis [38], although entire ventral hippocampus lesions did not [71].

Kallai et al. [81] assessed thigmotaxis during an incidental spatial memory task in a real, 6.5 m diameter arena, and in a virtual reality arena. In *N* > 100 participants, thigmotaxis correlated with self-reported fear [82] during early trials in both tasks, but not with trait anxiety [83]. Walz et al. [84] instructed participants to perform a free solitary walk on a real soccer field (Fig. 1B and Table 1). Thigmotaxis was increased in agoraphobia patients compared to healthy controls, and in those with high as compared to low self-reported anxiety sensitivity [85]. In a virtual reality version in three samples with overall *N* = 141, there was no consistent relation of thigmotaxis with various self-report scales of anxiety and fear [86]. Another study using a real room [87], and one with a virtual reality room [77], did not report data relevant for validation. The first three setups are related to what is used in in-vivo, or virtual reality-based psychotherapy, to elicit agoraphobia symptoms in patients [88].

### Operant conflict tests

In operant conflict tests, animals learn to associate a rewarded action, such as water drinking in the Vogel task [89] or food-rewarded lever pressing in the Geller–Seifter task [90], with additional punishment. Variants can involve sequential or simultaneous presentation of several punishment or reward intensities and schedules [91, 92]. Benzodiazepines and GABAergic triazolobenzodiazepines, as well as some tricyclic antidepressants, consistently increase punished behaviour [93], whereas the effects of 5HT1A agonists and SSRIs are controversial [93].

Aupperle et al. [57] (Fig. 1C and Table 1) developed a third-person view computer task (termed ‘AAC decision test’ here) in which human participants move an avatar to decide between their chances of receiving a conflict outcome (aversive sound and image, coupled with virtual reward) versus a non-conflict outcome. Cross-species validity of this task rests on a macaque version, which pitted airpuff punishment against food reward, and was itself validated with diazepam [24]. In this macaque task, an involvement of ACC in behavioural control was found [25], including increased ACC fMRI signal in conflict vs. non-conflict trials [27]. Similarly increased ACC activation was found in the human paradigm by two different laboratories [27, 94]. In *N* = 95 participants, sex predicted anxiety-like behaviour [57]. Post-hoc tests showed that anxiety-like behaviour correlated with anxiety sensitivity traits in males, and with fun seeking traits in females [57]. A follow-up study in people with anxiety disorder, depression or substance abuse showed no task- or disorder-specific relation of behaviour with diagnosis [95].

Bach [40] (Fig. 1D and Table 1) developed a third-person view computer game (termed ‘AAC scoop & run test’ here) in which participants are instructed to press a key to collect a financial reward, under threat of being ‘caught’ by a virtual ‘predator’ and losing previously collected rewards. Participants tend to collect fewer rewards when potential loss is higher [40, 41]. This tendency is reduced in persons with surgical and acute inflammatory hippocampus lesions [18]. Furthermore, gamma oscillations in HC and theta HC-PFC coupling increase with predator probability [33], and hippocampus (particularly CA2/3) BOLD signal increases with increased avoidance [39]. In *N* = 41 participants, self-reported trait anxiety [83] was related to behaviour in a non-linear way [40].

### Mixed open field and operant conflict test

Combining the open field and operant conflict test, Bach et al. [58] (Fig. 1E and Table 1) developed a third-person view computer game (termed ‘AAC stay & play test’ here) in which participants collect financial rewards and avoid a potentially ‘attacking predator’ (the operant component) by moving on a 24 × 16 grid (the open field component). Participants tend to explore and collect rewards early on and then retreat to a safe place as time progresses. The GABAergic anxiolytics lorazepam [17] and pregabalin [11] as well as degenerative hippocampus lesions [58] reduced this tendency to return to safety. Valproate, which has GABAergic properties and reduces anxiety-like behaviour in rodents [11], also reduced retreat to safety in this model [11]. BOLD signal in ventral hippocampus increased with probability of predator attack [58]. A large adolescent study (*N* = 781, *N* = 567 for 2-year test–retest reliability) demonstrated relatively stable individual differences in cautious behaviour [96]. Behaviour was best predicted by sex, IQ, self-reported daringness [97] and impulsivity [98], and had no relation to self-reported anxiety [99]. Notably, self-reported daringness does not correlate with self-reported anxiety [96] or neuroticism [97]. Thus, this test appears to measure stable individual differences in anxiety-like behaviour, which are however unrelated to self-reported anxiety.

### Learned or instructed unavoidable shock anticipation

When rodents are exposed to contexts associated with unpredictable and uncontrollable foot shocks, they come to express increased freezing [100], enhanced startle reflex [101] and will, if given a chance, avoid that context [102]. While observable behaviour is somewhat similar to what is elicited by fear-conditioned cues, the underlying neural control is largely different [103, 104]. In general, benzodiazepine effects on startle reflex are challenging to assess due to their muscle relaxant properties [104], but there is indirect [105] and direct [106] evidence for context-specific startle reduction under the benzodiazepine chlordiazepoxide. Several experiments showed that corticotrophin-releasing factor (CRF) antagonists reduce context-potentiated startle as well [104]. Since this paradigm involves contextual learning (and thus, creation of a longer-lasting phenotype as in common PTSD models [45, 46]), it is important to note that the studies discussed focus on the short-term expression of behaviour during and immediately after learning.

Grillon and Davis [107] (Fig. 1F and Table 1) translated this paradigm to humans, with three different conditions: no-shock, predictable shock and unpredictable shock. In this so-called NPU test, context-specific behaviour (usually startle amplitude) is quantified by normalisation to the no-shock condition, and then compared between predictable and unpredictable conditions. Context-specific startle potentiation is found in this [107] and other variants of the paradigm [108], as well as when US anticipation is verbally instructed rather than conditioned [109]. Furthermore, humans show behavioural avoidance of the context [108]. Most of the human studies discussed in the following use a verbally instructed version of the paradigm. Contextual startle potentiation (and additionally baseline startle) is reduced after treatment with 1 mg alprazolam [110, 111] but not 0.5 mg alprazolam or the sedative (non-anxiolytic) histamine-1 receptor antagonist 50 mg diphenhydramine [110]. It is also reduced by chronic SSRI treatment in healthy individuals [112]. A CRF antagonist (which has not been tested in clinical application) had no effect on contextual startle potentiation [111]. See [10] for a summary of the effects of further experimental compounds. In terms of diagnostic application, patients with PTSD [113], social anxiety and specific phobia [114] and panic disorder [115, 116], but not GAD [113, 114], or MDD [116], show increased contextual startle potentiation, compared to healthy individuals. In a large (*N* = 258) sample of trauma-exposed people, posttraumatic symptom severity linearly related to contextual startle potentiation [117].

### Social intrusion in children

Social intrusion tests differ from the other paradigms discussed here in two respects: first, they were initially developed in humans and then translated to monkeys; second, they were designed to measure individual differences, rather than group-level effects as the other tests. When confronted with novel objects or unfamiliar humans, children systematically differ in how shy or inhibited they behave [118]. This disposition—termed behavioural inhibition—can be detected as early as 4 months of age and is relatively consistent across situations and time points during childhood (see for review [119]). Behavioural inhibition is partly heritable [120], and is a significant risk factor for later development of social anxiety disorder [121]. Notably, there is no universally agreed behavioural paradigm to measure behavioural inhibition; however, many paradigms involve confrontation with an unfamiliar human [121]. Because this research is done in children, there is a dearth of data on the neurophysiology of the acute inhibition behaviour, or on anxiolytic treatments.

Kalin et al. [122–124] developed a monkey test in which a human confronts an infant monkey without eye contact, with the goal of maximising similarity of the observed behaviour between human children and monkey infants (i.e., face validity). Like humans, monkeys systematically differ in their behavioural (freezing, cooing) and endocrine response (CRF secretion) to this situation, and these differences are relatively stable over time [123]. The acute behavioural inhibition in this test is reduced by diazepam [122] and increased by the anxiogenic substance beta-carboline [125]. A plethora of research has addressed the neural control of behavioural inhibition, and in particular of individual differences, in monkeys (see for review [123, 124]). However, there is a lack of data sources that are directly comparable between the species [126].

## Synopsis

### Cross-species translation and neurobiological species differences

Seven distinct human anxiety tests have been directly validated across species (see Table 1 for details). In four of these paradigms (EPM, open field test, NPU test, social intrusion test), the behaviour-eliciting procedure and the resulting behaviour are more or less directly comparable across species, although the implied threats are necessarily and conceptually different. Two of these tests were further validated with benzodiazepines (EPM, NPU test). The other three paradigms are third-person view computer games, which constrains face validity. For one of these, a direct correspondence of human and monkey physiology has been demonstrated (AAC decision test), a second was validated with a benzodiazepine, pregabalin and clinical hippocampus lesions (AAC stay & play test), and the third with clinical hippocampus lesions only (AAC scoop & run test). Across paradigms, these validation data demonstrate some level of cross-species similarity in the systems-level neural control of behaviour in these tests. However, even though we have intentionally focused on those paradigms that were successfully validated in this way, notable species differences have been observed as well. For example, CRF agonists impact on behaviour in rodent [104] but not human NPU test [111], and hippocampal theta oscillation power relates to threat in rodent open field test and EPM [29], but not human AAC scoop & run test [33].

### Within-species drug screening

In view of such species differences in the neural control of behaviour, a candidate anxiolytic may fail in clinical translation simply because the targeted mechanism is relevant for rodent but not human avoidance behaviour. Such obstacles could potentially be detected more quickly and cost-effectively in human pre-clinical tests than in large phase II trials [10, 11]. The rationale is that a substance that has demonstrated anxiolytic potential in non-human tests, but is not anxiolytic in the healthy human test, is unlikely to be a successful clinical treatment.

Three paradigms appear potentially suitable for this purpose (EPM, AAC stay & play test, NPU test), as they their predictive validity has been established with benzodiazepines and at least one additional substance with known anxiolytic or anxiogenic properties (see Table 1 for details). Using this approach, two candidate anxiolytics were confirmed in the human NPU test. One of them (group II mGlu2/3 receptor agonist LY354740) was taken to a phase II trial with mixed results; development was halted, partly due to side effects [10]. In contrast, CRF agonists, which appear anxiolytic in rodents, had no effect in humans, and they also turned out to be clinically ineffective [10]. This supports the idea that pre-clinical screening could identify unsuccessful candidate drugs. In the AAC stay & play test, one candidate anxiolytic (valproate) was confirmed but not yet taken to a phase II trial [11].

Overall, the available data support the potential of this research strategy. However, there is no evidence yet that any phase II trials were motivated by, or abandoned due to, pre-clinical tests in humans. Indeed, this strategy has only been used by academic investigators who had developed and promoted a particular cross-species paradigm in the first place (including the author of this review), which may limit its credibility. In order to be a successful drug development strategy, industry and public funders would have to be convinced of its validity and cost-effectiveness. Establishing the latter may require a clearer view on the costs of pre-clinical trials, which are dictated by achievable effect sizes. These can be computed from behavioural variability in (untreated) control groups [127, 128], and define statistical power for a human screening trial. Some promising steps into this direction have been taken for the NPU task [129]. In conclusion, we suggest systematic and independent comparison of the predictive validity of different paradigms, and of their achievable effect sizes.

### Diagnostic tests

As a second application, if anxiety tests are sensitive to induced anxiety symptoms, then they may be developed into diagnostic or psychometric instruments. This could be motivated by evidence that they (a) measure stable individual differences, (b) differentiate clinical groups, (c) afford individual diagnosis, (d) prospectively predict anxiety disorder, (e) predict the course of the disorder including treatment responses and (f) have good psychometric properties. Some tests show potential on several of these criteria, but they are, for different reasons, still far away from clinical application.

Most prominently, stable individual differences of children and adolescents in social intrusion tests are well replicated, and constitute an established prospective risk factor for the development of social anxiety disorder [121]. This could potentially be useful for early detection and prevention programmes. However, studies diverge widely in the assessment of social intrusion responses [121]. Due to a lack of large samples for any particular instantiation of the test, it is not possible to quantify its psychometric or predictive properties.

Next, the NPU test has been established, on the group level, to distinguish PTSD, social anxiety disorder, specific phobia and panic disorder, from GAD, MDD, and healthy controls. It may also differentiate between individuals, since it relates to severity of PTSD symptoms [117]. However, this promising finding has not yet been replicated or extended.

For the other tests, their potential is less obvious. Open field test distinguishes clinical groups, and EPM is sensitive to anxiogenic drugs, but it is unknown whether they differentiate individuals. The AAC stay & play test measures stable individual differences but for all three AAC tests, investigation in clinical populations or with anxiogenic drugs is lacking. Interestingly, none of the AAC tests has a replicated relation with self-reported anxiety. This raises a possibility that cautious behaviour in these tests, and feelings of anxiety, are generated by unrelated processes. Since anxiety disorders usually involve feelings of anxiety, which accompany clinical avoidance behaviour, this might limit the clinical potential of the tests.

In conclusion, we suggest creating one (or several age-adapted) standardised version of the social intrusion test for children and adolescents, and validating its psychometric properties and predictive value in larger samples. For testing adults, we suggest combining several of the other tests into one battery, and directly comparing their psychometric properties and relation to diagnosis or clinical outcomes on the individual level.

### Aetiology of anxiety disorders

The third promise we reviewed is that behaviour of healthy individuals in the test may rely on the same physiology as anxiety symptoms in patients. If this was the case then physiological investigation in animal anxiety tests could potentially elucidate the aetiology of anxiety symptoms and neurobiological targets for symptom control. This idea has been debated for decades [5, 9, 13]. As yet, it rests on the observation that GABAergic substances reduce anxiety-like behaviour in the tests as well as anxiety symptoms in patients. One difficulty in finding additional empirical evidence is that few neurophysiological phenomena are actually established in anxiety disorders [1]. As a potential way forward, a recent meta-analysis compared fMRI signal between anxiety patients and healthy people during exposure to salient stimulus material. Differences were found in various brain areas including anterior cingulate, insula, amygdala and hippocampus [130]. Notably, this overlaps with brain areas in which fMRI responses during several anxiety tests discussed here have also been observed: hippocampus/amygdala response in the AAC stay & play test [58] and the NPU test [131] (although not in  a meta-analysis of unpredictable threat responses across various procedures [130]), anterior cingulate and insula in the NPU test [131] (as well as in the meta-analysis [130]) and ACC—across species—in the AAC decision test [27, 94]. While the general approach is promising, this meta-analysis compared patients and controls across many different tasks rather than only in symptom-provocation tasks. Hence, the specific role of these regions in symptom generation remains to be determined. Also, it would be desirable to corroborate fMRI results with observations that allow inference on faster time scales and thus better comparability to non-human neuroscience methods. New developments in EEG artefact control [132] and wearable MEG [133] could facilitate research in behaving patients, and single-unit recordings in pre-surgical epilepsy patients [134] allow direct comparison with animal studies. All of this may open exciting avenues for clinical research. A complementary way forward could be to harness existing anxiety tests to develop more standardised symptom-provocation tasks. These could facilitate investigating the neurophysiology that underpins symptoms in patients. However, at the moment and after decades of research, it appears unavoidable to concede at least a possibility that adaptive avoidance behaviour in healthy individuals—in the cross-species tests discussed here—might be controlled by neural mechanisms that are largely different from those that control or maintain anxiety symptoms in patients.

## Conclusions and future directions

We scrutinised several behavioural anxiety tests that have been translated across species boundaries. There is a lack of clinical innovation from rodent anxiety tests, and cross-species translation might help to identify potentially underlying neurobiological species differences. In addition, three clinically relevant goals could be achieved with these tests. The first is anxiolytic candidate drug screening in humans, to avoid clinical trials with compounds that are likely to fail due to species differences. Two tests (NPU and AAC stay & play test) have been used for this purpose but only by the academic investigators who developed them. Independent comparison of achievable effect sizes and thus statistical power of these tests, as well as of predictive validity, might be a way forward to convince industrial and public funders that this approach is useful and cost-effective.

A second goal is the development of diagnostic or psychometric instruments. Two tests (social intrusion and NPU test) have provided some evidence that they measure clinically relevant individual differences. However, for different reasons, both tests require further confirmation of the predictive value of test scores, and of their psychometric properties. Such investigation would usefully include other anxiety tests to maximise the innovation potential of a large clinical trial. Interestingly, such instruments could also be used to measure anxiety symptoms elicited in the various anxiety models that induce longer-lasting phenotypes [4, 7] and are not covered in this review.

The third goal is to reject or confirm the conjecture that anxiety tests are aetiologically relevant. Here, the hope is that they can help elucidating the neurobiological mechanisms that underly symptoms of anxiety disorder. At the minimum, this would require evidence of gross overlap in the neural control of avoidance behaviour during symptom provocation in anxiety disorder, and during these tests in healthy persons. However, beyond the action of a fairly restricted class of anxiolytic drugs, such evidence does not exist. We have highlighted several ways forward, including more research on the neurophysiological underpinnings of anxiety symptoms in controlled symptom-provocation tasks. However, at the moment, one possible conclusion is that the physiological mechanisms might indeed be different. Crucially, this would not invalidate the other two goals. The tests may still measure clinically relevant behaviour when applied to patients or at-risk persons, and thus be diagnostic. They may also be able to identify relevant drugs that act on the GABAergic and serotonergic systems—which we know are relevant both for anxiety symptoms and for behavioural avoidance in healthy people—and thus have a useful place in the drug development pipeline.

To summarise, further work is needed to render cross-species anxiety tests clinically relevant. However, even if the promise of aetiological insights into anxiety disorders may be unrealistic, they have led, and will continue to lead, to fascinating insights into the control of avoidance behaviour in healthy individuals, and thus contribute to our understanding of biologically relevant behaviour.
